# The role of aldosterone in the pathogenesis of diabetic retinopathy

**DOI:** 10.3389/fendo.2023.1163787

**Published:** 2023-04-11

**Authors:** Kangcheng Liu, Hua Zou, Huimin Fan, Hanying Hu, Yanhua Cheng, Jingying Liu, Xiaojian Wu, Bolin Chen, Zhipeng You

**Affiliations:** ^1^ Jiangxi Clinical Research Center for Ophthalmic Disease, Jiangxi Research Institute of Ophthalmology and Visual Science, Affiliated Eye Hospital of Nanchang University, Nanchang, China; ^2^ Hunan Key Laboratory of Ophthalmology, Eye Center of Xiangya Hospital, Central South University, Changsha, Hunan, China

**Keywords:** aldosterone, mineralocorticoid, diabetic retinopathy, inflammation, angiogenesis

## Abstract

Aldosterone, as a mineralocorticoid of adrenal origin, has effects that are not limited to the urinary tract. As an important regulator in Vasoactive hormone pathways, aldosterone may play an effect in the pathogenesis of diabetic retinopathy (DR) through the regulation of oxidative stress, vascular regulation, and inflammatory mechanisms. This implies that mineralocorticoids, including aldosterone, have great potential and value for the diagnosis and treatment of DR. Because early studies did not focus on the intrinsic association between mineralocorticoids and DR, targeted research is still in its infancy and there are still many obstacles to its application in the clinical setting. Recent studies have improved the understanding of the effects of aldosterone on DR, and we review them with the aim of exploring possible mechanisms for the treatment and prevention of DR.

## 1 Introduction

Diabetic retinopathy (DR) is a serious complication of diabetes that often present with no symptoms in the initial stages. However, if left untreated, hyperglycemia-induced retinal damage can lead to irreversible vision loss, which poses a serious threat to global health. Epidemiological studies reveal that up to 33% of diabetic patients suffer from diabetic retinopathy, with 18.89% of patients progressing to proliferative diabetic retinopathy (PDR), while 23.33% experience diabetic macular edema (1). Notably, these two conditions are the primary causes of irreversible blindness among working-age individuals.

Mechanistically, diabetic macular edema is induced by the high glucose environment, which not only destroys pericytes but also damages the blood-retinal barrier, causing retinal hemorrhage, protein leakage, and hard exudation (2). This persistent damage can further lead to the accumulation of leukocytes in acellular capillaries and microvessels, resulting in tissue ischemia and non-perfusion, which in turn promote retinal neovascularization (3). As diabetic retinopathy advances, patients’ vision may continue to decline, significantly impacting their overall well-being and quality of life (4, 5). Although anti-VEGF drugs and corticosteroids have shown some therapeutic benefits for DR treatment, they cannot halt disease progression. Therefore, current research should focus on the regulation of systemic factors (6).

**Figure 1:**
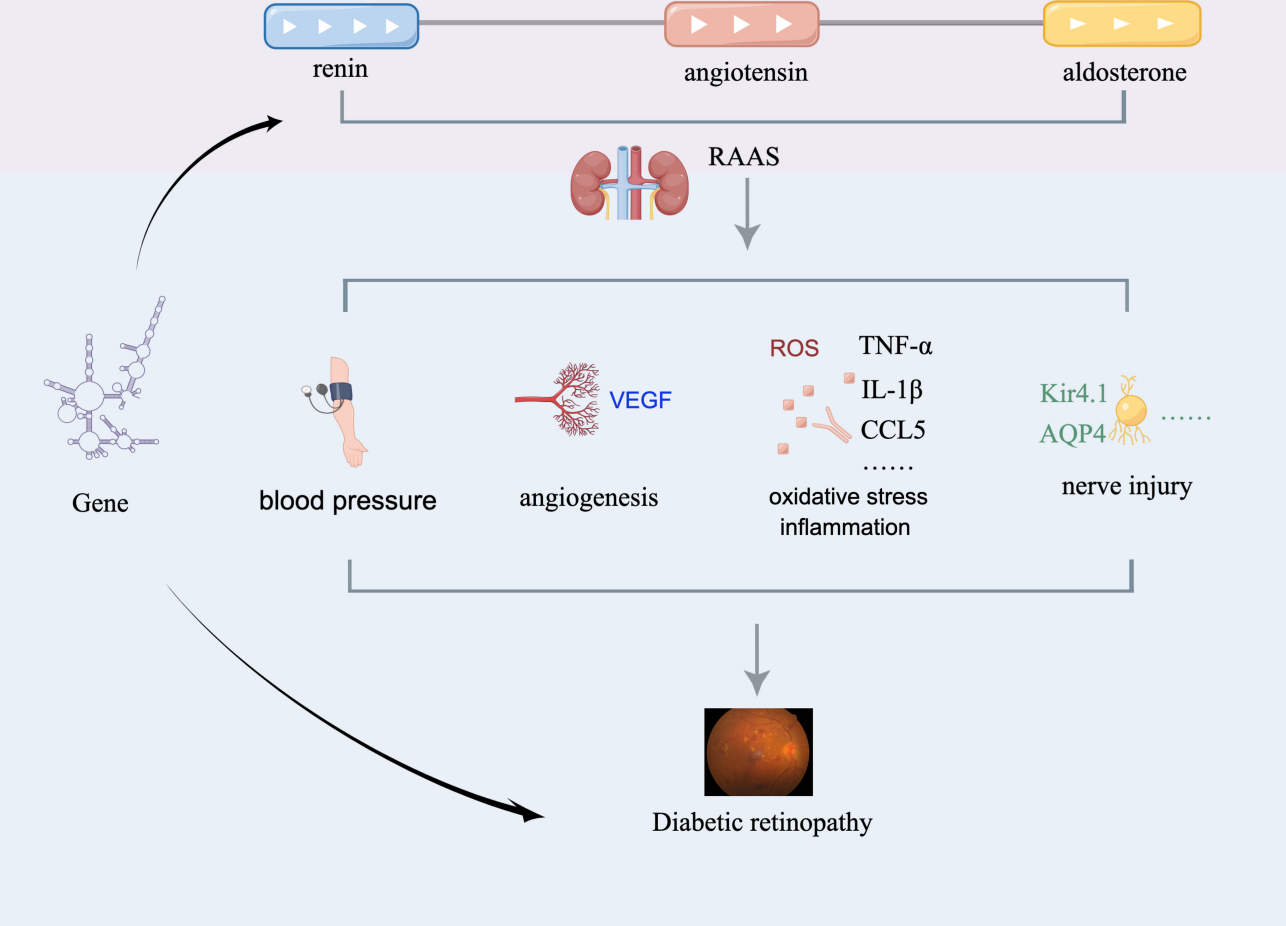
Putative mechanism whereby aldosterone affects DR. RAAS, renin-angiotensin-aldosterone system; AQP4, aquaporin-4; Kir4.1, inward rectifier potassium channel 4.1.

Mineralocorticoids, including aldosterone and deoxycorticosterone, are primarily secreted by cells in the zona glomerulosa of the adrenal cortex. Importantly, they regulate the concentration of potassium and sodium in the body through mineralocorticoid receptors (MRs) to maintain water and electrolyte homeostasis. The renin-angiotensin-aldosterone system (RAAS) is a critical part of the vasoactive hormone pathway, in which aldosterone plays a key role. When RAAS is stimulated and activated, angiotensin II stimulates adrenal glomerular cells to release aldosterone, which then causes a series of pathophysiological changes such as tissue inflammation (7), fibrosis (8) cell proliferation (9), oxidative stress (10) and neovascularization (11–13) (**Figure 1**). Based on these findings, researchers confirmed that aldosterone is critical in the pathological mechanism of cardiovascular disease and chronic kidney disease (14). It is worth noting that glucocorticoid receptors are widely present in the retina (15). Wilkinson-BerkaJL et al. (15) previously found that activation of the glucocorticoid receptor adversely affects the retina by promoting inflammation and fibrosis. Allingham et al. (16) also uncovered that RAAS has a local effect on the retina, contributing to the occurrence and progression of DR. Meanwhile, Lovshin et al. (17) discovered that retinal vascular hardness could increase in DR patients after RAAS activation, implying that RAAS components have a fibrotic effect. Collectively, these studies provide theoretical support for aldosterone as a target for the treatment of DR.

Nonetheless, upon reviewing previous studies on the relationship between mineralocorticoids and DR, few investigations have specifically focused on aldosterone and DR pathogenesis. Therefore, this review will primarily focus on the significance of aldosterone as a mineralocorticoid in the onset and progression of diabetic retinopathy, aiming to provide direction for future DR prophylaxis and treatment strategies.

## 2 Effect of aldosterone-mediated blood pressure changes on diabetic retinopathy

The effect of RAAS was discovered and described earlier in the study about the urinary system. In the study of Ames et al. (18), components of the RAAS signaling pathway were found not only in the kidney but also in the heart, brain, lung and eyes, and various cells of the retina. Interestingly, Lai et al. (19) previously surmised a relationship in terms of common pathogenic mechanisms between kidney and eye diseases. The key link between kidney and eye diseases lies in inflammation, oxidative stress and other injuries caused by RAAS disorder. Lai et al. (19) observed that RAAS affects both the glomeruli and retina in similar ways. In the eye, RAAS plays a role in neovascularization and maintaining aqueous humor circulation stability. Additionally, RAAS can influence the blood flow in the ciliary body, iris, and retina, and strictly regulate intraocular pressure by modulating the production and excretion of aqueous humor. Furthermore, RAAS also participates in the development of retinal exudation and macular edema.

In a case-control study conducted by Senanayake et al. (20), the retina of diabetic patients was found to have a higher level of angiotensin II compared to nondiabetic patients. The study also detected higher levels of angiotensin II in the vitreous fluid of DR patients compared to non-diabetic patients (21), indicating that RAAS plays a critical role as a mediator of DR occurrence and progression. In a study by Lovshin et al. (17) involving long-term observation of 75 patients with type 1 diabetes, it was revealed that the angiosclerosis process in patients with PDR might trigger the continuous activation of the renin-angiotensin-aldosterone system. Notably, the positive role of RAAS in the regulation of blood pressure (BP) is well established. Pathological changes in RAAS can result in elevated BP, which is a significant risk factor for diabetic retinopathy. Some researchers suggest that patients using RAAS antagonists may lower their BP and thus reduce the risk of DR, primarily through the use of angiotensin-converting enzyme inhibitors and angiotensin receptor blockers (22). This observation is consistent with findings from rodent models, whereby RAAS antagonists were found to prevent retinal capillary leakage and reduce the risk of DR in diabetic animal models (23). These benefits were likely mediated indirectly by lowering blood pressure.

Aldosterone plays a role in the development of diabetic retinopathy by regulating blood pressure changes. Although most studies on this topic are still based on animal models, some inhibitors of RAAS components have shown promise in treating DR in such models. However, the safety and efficacy of aldosterone antagonists in the clinical therapy of DR need to be further investigated in human subjects.

## 3 Effect of aldosterone-mediated angiogenesis on diabetic retinopathy

It is worth noting that current studies have found that aldosterone has a certain effect on neovascularization. As a key molecule of RAAS, aldosterone binds to its corresponding MR to promote angiogenesis. Zhao et al. (24) observed an increase in endothelial growth factor mRNA and neovascularization in rat that received aldosterone injections, suggesting a potential role of aldosterone in promoting DR progression by elevating vascular endothelial growth factor (VEGF) levels and retinal neovascularization. Although the underlying mechanism is not fully understood, other research results support this finding.

Müller cells express high levels of mineralocorticoid receptors and secrete large amounts of VEGF, which contributes to neovascularization in DR (25). In a study investigating the effect of aldosterone on retinopathy of prematurity, researchers found that aldosterone could aggravate retinal neovascularization. However, interventions with spironolactone (an MR inhibitor) and an aldosterone synthase inhibitor were shown to effectively reduce retinal angiopathy and inflammation, as well as prevent retinal neovascularization by reducing VEGF levels and inflammatory factors (26).

The increase in VEGF is a well-known factor leading to diseases such as DR and retinopathy of prematurity (27). Feng et al. (28) observed the effects of KCTD10 on DR and animal models and discovered that reducing VEGF improved cell vitality and alleviated DR symptoms in rats. This finding further supports the critical role of VEGF in the development of DR. Furthermore, studies have shown that RAAS antagonists can attenuate the endothelial cell barrier dysfunction induced by VEGF, suggesting that these agents may enhance the response of DR patients to VEGF (29). It is interesting to note that aldosterone may be involved in the expression of VEGF and contribute to the development and progression of DR. Additionally, previous studies have highlighted the significant involvement of advanced glycation end products (AGEs), receptors for AGE, and nuclear factor erythroid 2-related factor 2 (NRF2) in the pathogenesis and progression of DR (30, 31). Kang et al. (32) demonstrated that AGE significantly increases VEGF mRNA expression in cells, indicating a positive correlation between AGE and VEGF levels. Moreover, recent research on diabetic nephropathy revealed that blocking receptors for AGE reduces aldosterone’s negative impact on NRF2 signal mediators in renal cells, suggesting that aldosterone may block the binding of AGE and RAGE to reduce VEGF production and affect NRF2, leading to corresponding harm to the retina (33). Therefore, aldosterone may play a role in regulating angiogenesis through its effect on VEGF, thus playing a therapeutic role in DR.

MR has been identified in various retinal cells, such as vascular endothelial cells, pericytes, neurons, ganglion cells, and retinal pigment epithelial cells, indicating that aldosterone can activate MR in these target tissues (34). However, the exact mechanism of this activation remains elusive. Notably, although cortisol levels in the serum are 100 times higher than aldosterone levels, MR has a similar affinity for both hormones. Indeed, the specific binding ability of aldosterone to MR and its downstream signaling pathways are important issues that warrant further investigation. Some researchers have postulated that aldosterone may express an enzyme that inactivates cortisol, allowing it to bind to MR (16). However, the exact mechanism by which aldosterone specifically binds to MR and its downstream effects in diabetic retinopathy remain poorly understood. Despite some progress in understanding its role in angiogenesis, further investigation is needed to elucidate the specific contribution of aldosterone in the development and progression of diabetic retinopathy.

## 4 Effects of aldosterone-mediated oxidative stress and vascular inflammation on diabetic retinopathy

In patients with diabetes, Kang Q et al. found that the excessive production of reactive oxygen species (ROS) in the retina, and the accumulation of ROS due to insufficient clearance by the antioxidant defense system, are key factors in the development of diabetic retinopathy (35). RanaI et al. demonstrated that reducing aldosterone synthase and mineralocorticoid receptors in retinal microglia resulted in reduced intracellular ROS, indicating that local aldosterone in the retina may stimulate the overproduction of ROS, leading to oxidative stress and retinal injury (36). These findings suggest that targeting aldosterone and MR may be a potential therapeutic strategy for preventing or treating diabetic retinopathy. In addition to promoting the accumulation of intracellular ROS, persistent elevation of aldosterone can also block the synthesis of nitric oxide and activate cyclooxygenase-2 (37, 38). While some kidney-focused clinical studies suggest a deeper perspective, Higashide T et al. found that ROS accumulation can also promote the increase of aldosterone in patients with aldosteronism and eye diseases (39). However, Reina-Couto et al. (40) suggested that a high level of aldosterone in the blood may inhibit the contraction of retinal veins due to the high inflammatory response of blood vessels. As the specific mechanism has not been fully elucidated, further studies are required to confirm these findings.

Nevertheless, aldosterone has been found to promote inflammatory damage to the retina by inducing the production of pro-inflammatory factors such as interleukin (IL)-1β, CCL5, and TNF-α. Studies by Dong et al. (41) have demonstrated the up-regulated expression of adhesion molecules in the retina of diabetic rats, including vascular cell adhesion molecule-1 in retinal endothelium, which leads to leukocyte adhesion and plays a critical role in retinal angiogenesis and the development of proliferative diabetic retinopathy. Inhibition of aldosterone synthase by FAD286 has been shown to effectively reduce the expression of pro-inflammatory factors such as TNF-α and vascular cell adhesion molecule-1 in the retina (26). Studies by Tang et al. (42) and Kong et al. (43) have also revealed increased expression of inflammatory cytokines such as IL-1β and TNF-α in DR, leading to an inflammatory response that promotes the development of DR. Thus, aldosterone may mediate the expression of cell adhesion molecules and TNF-α, to exacerbate retinal vascular inflammation. Therefore, understanding the regulation of aldosterone on inflammation has potential therapeutic value and research significance in the prevention and treatment of DR.

## 5 Effect of aldosterone-mediated nerve injury on diabetic retinopathy

The pathophysiology of DR involves not only angiogenesis and inflammatory injury but also neurodegeneration. Müller cells are present throughout the retina and surround the neurons and microvessels, playing a crucial role in maintaining retinal structure and homeostasis. In addition, müller cells secrete neurotrophic factors that protect retinal nerve cells, making them vital for retinal health (44, 45).

Hyperglycemia-induced stimulation of retinal müller cells leads to an increase in intracellular inflammatory factors, VEGF, and chemokines, which promote the development and progression of DR. Interestingly, müller cell activation has varying effects on the retina depending on the stage of DR. During the non-PDR phase, activation of müller cells can protect retinal nerve cells, reduce edema and maintain the integrity of the retina (44). However, in the PDR stage, müller cell activation can promote retinal cell apoptosis, weaken the blood-retinal barrier and increase the production of inflammatory factors (46). These findings highlight the important role of müller cells as the main mediator of retinal inflammation and vascular leakage in DR.

Zhao et al. (34) have previously conducted studies that show that aldosterone can regulate the ion/water pathway of retinal Müller cells, thereby maintaining normal retinal function. This finding is further supported by Allingham et al., who suggested that aldosterone can influence the expression and localization of aquaporin-4 (AQP4) and inward rectifier potassium channel 4.1 (Kir4.1) in müller cells, leading to the accumulation of glial fibrillary acidic protein (GFAP), a marker of müller cell damage (47). AQP4 and Kir4.1 are responsible for the entry and exit of water and potassium ions in müller cells, mediate the humoral transport of müller cells and maintain the balance of water and electrolytes in müller cells (48, 49). Similarly, the accumulation of water and potassium in müller cells will lead to DR aggravating retinal degeneration. The upregulation of AQP4 and downregulation of Kir4.1 can disrupt the balance of water and electrolytes in müller cells, leading to the accumulation of water and potassium, which exacerbates retinal degeneration and promotes the development of DR. In addition, these changes can stimulate the release of inflammatory factors like IL-1 β, IL-6, IL-17, and TNF- α, as well as the production of growth factors such as VEGF, further promoting the occurrence and progression of DR (50). Deliyanti et al. (26) conducted studies that confirmed the presence of aldosterone synthase, mineralocorticoid receptor, and 11-hydroxysteroid dehydrogenase type 2 in müller cells, indicating that aldosterone can specifically react with müller cells in DR patients. As a result, aldosterone inhibitors can be used to target the water and electrolyte transport pathways in müller cells. However, further research is required to validate the feasibility of this approach.

## 6 Evaluation of the effect of aldosterone on DR from the perspective of gene

From the perspective of genetics, many scholars have also evaluated the association between aldosterone and DR at the genetic level. Ohashi et al. (51) treated müller cells with high glucose. MR inhibitors inhibited aldosterone-induced cell swelling and up-regulated MR target genes. Younas et al. (52) conducted a genetic polymorphism study on G8790A related to RAAS, and found that A genotype (male) and AG/AA genotype (female) are risk factors for diabetes, which did not confirm the association with DR. However, in the study of Egyptian cases, MTHFR 677 TT, MTHFR 1298 CC, AC and ACE DD genotype were found to be related to DR (53). It is worth noting that the conclusion of the study is relatively limited due to the interference of race, sex, age and other factors in the study of gene level. There are many difficulties in the research of large samples and multi-ethnic. Therefore, to explore the effect of aldosterone on DR from the perspective of genetics, it is still necessary to balance more and more confounders in order to obtain more reliable conclusions.

## 7 Conclusion and prospect

With an increased understanding of the role of aldosterone in DR, treatments targeting various pathways and molecules have emerged. Aldosterone, as a component of RAAS, can induce retinal neovascularization and inflammation, both of which are linked to the development of DR. The use of aldosterone and its inhibitors in the treatment of diabetes and its complications has garnered significant interest, and many studies have explored various methods of aldosterone inhibition in DR treatment. However, there is still limited knowledge regarding the mechanisms underlying these effects. Further investigation into the role and mechanisms of aldosterone in DR could have significant clinical implications.

Currently, the impact of aldosterone on retinal damage is not fully understood in the field of ophthalmology. It remains unclear whether the damage is caused mainly by local or systemic factors, which limits the clinical use of aldosterone. Nevertheless, aldosterone antagonists have demonstrated some signs of efficacy in treating retinal diseases (24). Future research should use human eye tissues to determine the physiological and pathological role of aldosterone in DR, giving full play to the role of aldosterone antagonists in the treatment of patients with DR. Furthermore, the impact of different concentrations of aldosterone on the retina at different stages of DR needs to be explored, adding further complexity to the study of aldosterone, and opening up opportunities to discover new therapeutic targets.

## Author contributions

KL, HZ and ZY contributed to the conception of the review, and wrote the manuscript. All authors critically revised the manuscript. All authors contributed to the article and approved the submitted version.
